# Pruritus in atopic dermatitis: a cross-sectional study of adult patients from a tertiary university hospital in São Paulo, Brazil^☆^

**DOI:** 10.1016/j.abd.2024.09.006

**Published:** 2025-03-11

**Authors:** Georgia Biazus Soares, Raquel Leão Orfali, Beatriz Lacerda Averbach, Qai Ven Yap, Gil Yosipovitch, Valeria Aoki

**Affiliations:** a Dr. Phillip Frost Department of Dermatology and Cutaneous Surgery, University of Miami Miller School of Medicine, Miami, FL, USA; b Department of Dermatology, Faculty of Medicine, Universidade de São Paulo, São Paulo, SP, Brazil; c Biostatistics Unit, Yong Loo Lin School of Medicine, National University Health System, Singapore

**Keywords:** Dermatitis, atopic, Eczema, Pruritus

## Abstract

**Background:**

Chronic pruritus is the defining symptom of atopic dermatitis (AD). Although AD is common in Latin America, there is little data regarding pruritus intensity, characteristics, and effects on quality of life in this population.

**Objective:**

This cross-sectional study aimed to evaluate pruritus in 91 patients with AD at a tertiary university hospital in São Paulo, Brazil. Patients aged 14‒65-years old were included in this study.

**Methods:**

Patients completed the Itch Questionnaire, the ItchyQoL, and the POEM questionnaires and were asked to rate their itch severity using a 10-point peak pruritus Numerical Rating Scale (NRS). AD severity was assessed via the EASI and the vIGA-AD.

**Results:**

The mean age was 29.68 ± 12.87years, and 56.0% of patients were White. 97.8% of patients were currently experiencing pruritus with an average NRS of 7.32 ± 2.22. Patients had associated bleeding (71.4%), heat sensation (63.7%), and pain (54.9%). Worsening factors included stress (93.4%), dry skin (91.2%), and sweat (75.8%). The mean total ItchyQoL score was 78.93 ± 17.20. Female gender was significantly associated with a higher total ItchyQoL score (p = 0.009). Pruritus on the neck, foot, and whole body was associated with higher total ItchyQoL scores in adjusted models (p < 0.05). The EASI, vIGA-AD, and POEM were moderately correlated with itch intensity (*r* = 0.434, 0.406, and 0.610) and total ItchyQoL score (*r* = 0.425, 0.436, and 0.631).

**Study limitations:**

The predominantly White population cohort may not be representative of the diverse AD phenotypes in the Brazilian patient population. Children under the age of 14 and adults over the age of 65 were excluded from the population cohort. Furthermore, patients included in the study may suffer from other non-dermatological diseases that cause itch, which may influence the outcomes oberserved.

**Conclusions:**

Patients with AD in Brazil experience significant pruritus that impacts their quality of life. Gender, body location of itch, associated pain, and stress should all be taken into consideration when evaluating AD patients with pruritus.

## Introduction

Atopic Dermatitis (AD) is a chronic, relapsing inflammatory skin disease. Chronic pruritus, defined as pruritus of duration > 6-weeks, is one of the predominant and most bothersome symptoms of AD.^1^, ^2^ Itch significantly impacts patients’ Quality of Life (QoL) and mental health, and has been associated with impairments in work, sleep, and daily activities.^3^, ^4^, ^5^ Studies have reported a 20.1% prevalence of AD in Brazil, the highest among Latin American countries.^6^ However, there is a paucity of literature on the impact of AD-associated pruritus in Latin America, and itch intensity, characteristics, and effects on quality of life have not been well-studied in this population.

The primary objective of this cross-sectional study was to evaluate the prevalence and severity of itch in patients with atopic dermatitis at an AD clinic in a tertiary University hospital in Sāo Paulo, Brazil. The secondary objective was to describe the characteristics of pruritus in this patient population and its effect on Quality of Life (QoL). Furthermore, the authors aimed to assess the clinical severity of AD and its association with patient-reported pruritus measures.

## Methods

The authors recruited 91 patients with AD from the Atopic Dermatitis Clinic at the Department of Dermatology, University of São Paulo Medical School, São Paulo, Brazil from August 2022 to April 2023. Patients aged 14 to 65 years old diagnosed with AD according to the Hanifin and Rajka criteria were included in the study. Patients with AD who experienced pruritus were asked to complete three validated questionnaires including the Itch Questionnaire,^1^ the Itch-specific Quality of Life Questionnaire (ItchyQoL),^7^ and the Patient-Oriented Eczema Measures (POEM) questionnaire. Patients were also asked to rate their itch severity using a 10-point peak pruritus Numerical Rating Scale (pp-NRS).^8^ Patients were then fully examined by a dermatologist, and AD severity was assessed via 2 measures: The Eczema Area and Severity Index (EASI), and the validated Investigator Global Assessment Scale for Atopic Dermatitis (vIGA-AD). Informed consent was obtained from all participants and the study was approved by the local Ethics Committee.

Statistical analyses were performed using the Statistical Package for the Social Sciences, Version 29 (IBM Cooperation, New York). Continuous variables were presented as mean (SD), while categorical variables were presented as n (%). Unadjusted and adjusted linear regression was performed on the patient’s itch intensity and various scores. The Pearson correlation coefficient was used to investigate the relationship between patient’s itch intensity and each of the scores, and between the various scores.

## Results

**Figure 1:**
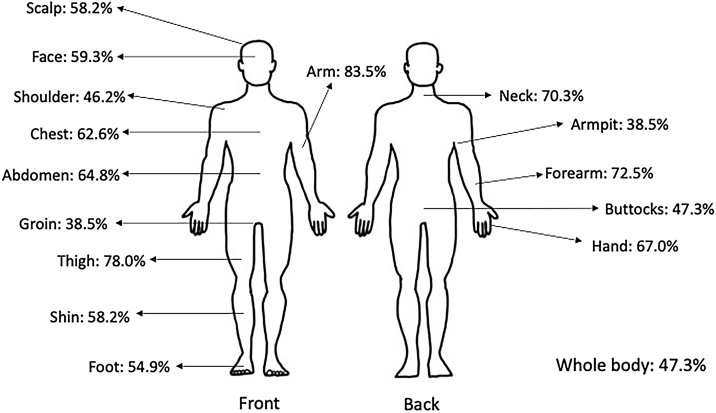
Pruritic body sites.

Demographic characteristics are listed in Table 1. The mean age was 29.68 ± 12.87 years, and the patient cohort was predominantly White (56.0%). Only 2 patients reported having another skin disease (1 psoriasis, 1 other- not specified). Itch characteristics are listed in Table 2. The majority of patients (97.8%) were currently experiencing pruritus, with an average peak pruritus NRS of 7.32 ± 2.22 in the last two weeks. Patients with pruritus had associated bleeding (71.4%), heat sensation (63.7%), and pain (54.9%). The most common itchy body sites included the arm, forearm, and thigh (Fig. 1). Worsening factors included stress (93.4%), dry skin (91.2%), and sweat (75.8%). The mean total ItchyQoL score was 78.93 ± 17.20, indicating that pruritus had a significant impact on quality of life. Mean scores for measures of eczema severity revealed that patients suffered from moderate to severe disease (Table 3).

**Table 1:** Demographic characteristics.

N = 91	n (%)	Mean (SD)
**Age**		29.68 (12.87)
**Sex**		
- Male	49 (53.8%)	
- Female	42 (46.1%)	
**Race**		
- Caucasian	51 (56.0%)	
- Asian	8 (8.8%)	
- Brown	26 (28.6%)	
- Black	6 (6.6%)

**Table 2:** Itch characteristics.

N = 91	n (%)	Mean (SD)
**Current pruritus, yes**	89 (97.8%)	
**Pruritus NRS in last 2-weeks**		7.32 (2.22)
**Associated symptoms**		
- Bleeding	65 (71.4%)	
- Heat sensation	58 (63.7%)	
- Pain	50 (54.9%)	
**Itchy body locations**		
- Arm	76 (83.5%)	
- Thigh	71 (78.0%)	
- Forearm	66 (72.5%)	
**Worsening factors**		
- Stress	85 (93.4%)	
- Dry skin	83 (91.2%)	
- Sweat	69 (75.8%)	

NRS, Numerical Rating Scale.

**Table 3:** Disease severity scores.

	Mean (SD)	Range
**POEM**	18.35 (6.94)	0‒28
**EASI**	20.87 (14.76)	0‒72
**vIGA-AD**	3.69 (0.90)	0‒4
**ItchyQoL**	78.93 (17.20)	22‒110

POEM, Patient Oriented Eczema Measures; EASI, Eczema Area and Severity Index; vIGA-AD, validated Investigator Global Assessment Scale for Atopic Dermatitis; ItchyQoL, Itch-specific Quality of Life Questionnaire.

The authors found that demographic characteristics such as age and gender did not have a statistically significant effect on pruritus intensity. However, Asian race was associated with a higher pruritus intensity (p = 0.036). While race and age did not have an effect on the total ItchyQoL score, female gender was significantly associated with a higher total ItchyQoL score (p = 0.009). Furthermore, race did not affect different measures of AD severity (Supplementary Tables 1‒5). The authors did not find a statistically significant association between assessed body locations and pruritus intensity in the adjusted models (Supplementary Table 1). Pruritus on the neck, foot, and whole body were statistically associated with higher total ItchyQoL scores in the adjusted linear regression model (p < 0.05) (Supplementary Table 2). Measures of AD severity including EASI, vIGA-AD, and POEM were moderately correlated with itch intensity (*r* = 0.434, 0.406, and 0.610, respectively) and total ItchyQoL score (*r* = 0.425, 0.436, and 0.631, respectively).

## Discussion

**Figure 2:**
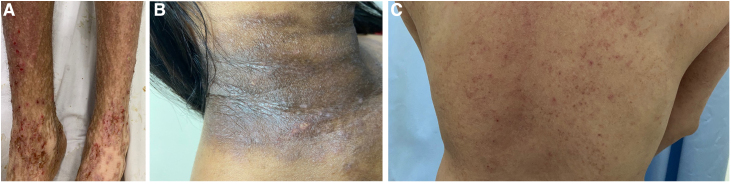
(A‒C) Characteristic features of atopic dermatitis in Brazilian patients.

The present results show that pruritus affected most patients with AD from a tertiary referral University hospital in São Paulo, Brazil, with an average moderate-to-severe intensity (Fig. 2A‒C). Large-scale studies in Europe and the US demonstrate that itch is the most burdensome symptom in AD patients, with a mean past week itch NRS score of 6.1.^2^ Results from one study evaluating the burden of AD in Brazil, Mexico, and Argentina found that severe pruritus ‒ defined as a worst pruritus NRS > 7 ‒ was reported by 54.4% of patients.^9^ The high pruritus prevalence and intensity in Brazilian patients with AD may suggest a need for better symptomatic control. A study evaluating disease management in AD patients in Brazil revealed that common treatment options included topical corticosteroids, emollients, systemic antihistamines, and oral corticosteroids. 79% of patients had discontinued or switched AD treatment, with 33% of those being due to poor effectiveness.^10^ Furthermore, a Brazilian report found that 42% of severe AD patients have more than 10 disease flares per year, with a median duration of 12-days.^11^ Dupilumab, a fully human Ig4 monoclonal antibody directed against the Interleukin-4 Receptor subunit alpha (IL-4Rα) of IL-4 and IL-13 receptors, is a biologic therapy that has revolutionized the treatment of AD, with landmark trials demonstrating its efficacy in significantly reducing pruritus.^12^, ^13^ Janus Kinase (JAK) inhibitors have also been increasingly used in patients with AD. By targeting the Janus-Kinase Signal and Activator of Transcription (JAK-STAT) pathway, these drugs downregulate inflammatory pathways and cytokines and therefore significantly reduce pruritus as well as AD severity.^14^ Although dupilumab and JAK inhibitors have been approved for use in many Latin American countries including Brazil and are recommended by the latest Brazilian Society of Dermatology consensus for use in patients with AD, accessibility remains limited due to factors such as cost and insurance coverage.^14^, ^15^

Bleeding, heat sensation, and pain were the most commonly reported associated symptoms with pruritus in the population cohort. Bleeding can occur secondary to scratching and reflects the intense pruritus experienced by these patients. Heat sensation and pain have been reported in previous studies assessing itch characteristics in AD.^1^, ^16^, ^17^ Skin pain, in particular, is a common finding in patients with AD, with one large population study showing that 42% of patients experienced both pain and pruritus on AD lesional skin.^2^ Patients with concomitant pain were more likely to describe their itch as tingling, burning, or stinging, characteristics that are classically associated with neuropathic pain.^18^ Pruritus and pain share common sensory neurons, mediators, and receptors, as well as similar mechanisms of neuronal sensitization.^19^ However, the relationship between these two sensations in the context of AD is not fully understood. Importantly, skin pain in AD strongly correlated with measures of AD severity and quality of life, highlighting the need to assess and treat pain in these patients.^18^

Most patients in this study reported that stress was an exacerbating factor for their pruritus, which is consistent with previous studies assessing itch in patients with AD.^16^, ^17^ Psychological stress is known to exacerbate AD symptoms via its effects on the hypothalamic-pituitary-adrenal axis, immune response, neuropeptide expression, and skin barrier function.^20^, ^21^, ^22^ Stress also exacerbates scratching, thus contributing to the itch-scratch cycle that aggravates the disease.^23^ Therefore, stress management should be addressed when treating patients with AD. A study from Brazil, however, found that only 11.8% of AD patients reported receiving therapy or psychological support.^10^

The authors found that the pruritus was most common on the arm, forearm, and thigh, and coincided with classic areas of AD lesions. After adjusting for other variables, pruritus on the neck, foot, and “whole body” was associated with a higher total ItchyQoL score, indicating a worst itch-related quality of life. Studies have shown an association between head and neck eczema and poor quality of life in AD patients.^24^, ^25^ This may be partially due to the fact that these areas are often visible.^25^

From the demographic factors evaluated in the present study, the female sex was significantly associated with a worse itch-related QoL. A previous study found that female sex was a predictor of poor itch-specific QoL in chronic pruritus patients.^26^ Furthermore, a positive correlation between Dermatology Life Quality Index (DLQI) score and disease severity in female patients with AD has been reported, although other studies did not find a meaningful gender difference in DLQI scores.^27^, ^28^ Interestingly, race was not significantly associated with a higher total ItchyQoL score or higher AD severity scores. Studies have shown that patients with skin of color are disproportionately affected by pruritus, and that non-White patients with chronic pruritus experience a greater impact on their quality of life.^29^, ^30^ Furthermore, AD is reported to be more common in patients with skin of color in U.S and Europe, and many genetic, immunologic, and clinical differences exist among various races and ethnicities.^30^, ^31^, ^32^, ^33^ The absence of significance in the present findings may reflect the admixture of the Brazilian population and the fact that race is self-declared, leading to a lack of Black and Asian patients in the population cohort. The authors did find that patients of Asian race (Fitzpatrick III) had a significantly higher pruritus intensity when compared to other races. However, this must be interpreted carefully due to the small number of Asian participants in this study. Given the diverse, heterogeneous population of Brazil, the authors suggest that the race and ethnicity of patients with AD should be taken into consideration when making diagnostic and therapeutic decisions.

The average total ItchyQoL score indicates that pruritus causes a significant burden and impacts the quality of life of these patients. The impact of pruritus on various aspects of QoL in patients with AD is well known.^2^, ^3^, ^34^ Furthermore, our results demonstrate that patient-reported measures of pruritus such as pp-NRS and ItchyQoL moderately correlate with objective measures of AD severity such as the EASI and vIGA-AD. Moderate correlations between the ItchyQoL and AD severity measures such as EASI and Scoring AD (SCORAD) have been previously reported. The authors concluded that this instrument could be incorporated into the assessment of AD patients and could be used to guide decision-making.^35^ Another study found that NRS worst itch and NRS average itch moderately correlated with EASI and objective-SCORAD, but not with vIGA-AD.^36^ The product of vIGA and body surface area (vIGA × BSA), however, has been shown to have weak-to-good convergent validity with NRS worst itch, NRS average itch, and ItchyQoL.^37^ The present findings indicate that itch can serve as an indicator of overall disease severity. Despite the significant QoL burden caused by pruritus, patient-reported itch outcomes in AD are not commonly evaluated using validated questionnaires in Brazil and other Latin American countries.^38^ The authors believe the current study highlights the importance of adding patient-reported measures of pruritus into regional consensuses on AD management and incorporating them into clinical practice.

### Limitations

The main limitation of this study is that the predominantly White population cohort may not be representative of the diverse Brazilian population and therefore may not accurately reflect all of the diverse AD phenotypes in the patient population. Children under the age of 14 were excluded from the population cohort, for the assessment of pruritus in children can be difficult and has limitations. This could, however, affect the overall prevalence of this study as AD is a common condition in children. Furthermore, patients over the age of 65 were excluded, as itching in the elderly can be multifactorial and can occur secondary to diagnoses other than AD. Patients included in the study may suffer from other non-dermatological diseases that cause itch, which may influence the outcomes observed in this study.

## Conclusion

In conclusion, patients with AD in Brazil experience significant pruritus that impacts their quality of life. Gender, body location of itch, presence of associated pain, and stress should all be taken into consideration when evaluating AD patients with pruritus, for these factors may contribute to the burden experienced by patients and could impact therapeutic decision-making. Furthermore, the authors found that patient-reported itch measures moderately correlate with other AD severity scores, demonstrating that these could be incorporated into the clinical evaluation of patients with AD. Evaluating for certain pruritic biomarkers present in the serum and skin, and correlating them to itch intensity and other characteristics in this population could provide us with more information to better tailor and personalize treatment.

## Financial support

Applebaum Foundation support to Gil Yosipovitch and Georgia Biazus Soares.

## Authors' Contributions

Georgia Biazus Soares: Investigation, original draft preparation.

Raquel Leao Orfali: Conceptualization, investigation, methodology.

Beatriz Lacerda Averbach: Investigation.

Yap Qai Ven: Formal analysis.

Gil Yosipovitch: Conceptualization, methodology, review and editing.

Valeria Aoki: Conceptualization, investigation, methodology, review and editing.

## Conflicts of interest

Raquel Leao Orfali has been an investigator and/or consultant for Bayer, Sanofi, Eli Lilly, Amgen; Valeria Aoki has been an investigator and/or consultant to Abbvie, Eli Lilly, Pfizer, Sanofi and Leopharma; Gil Yosipovitch has been Advisory Board Member for Abbvie, Arcutis, BMS, Cara Therapuetics, GSK. Escient Health, Eli Lilly, Galderma, Kiniksa Pharmaceuticals, LEO Pharma, Novartis, Pfizer, Pierre Fabre, Regeneron Pharmaceuticals, Inc., Sanofi, TreviTherapeutics, and Vifor; Receives grants/research funding from Eli Lilly, Kiniksa Pharmaceuticals, LEO Pharma, Novartis, Pfizer, Galderma, Escient, Sanofi Regeneron, and Celldex; Investigator for Regeneron Pharmaceuticals, Inc., Sanofi; Beatriz Lacerda Averbach is an investigator for Sanofi; Georgia Biazus Soares and Yap Qai Ven have no conflicts of interest to declare.
