# Supplementary material for: Pruritus in atopic dermatitis: a cross-sectional study of adult patients from a tertiary university hospital in São Paulo, Brazil
Source: An Bras Dermatol. 2025 Mar 11;100(4):101093. doi: 10.1016/j.abd.2024.09.006 (PMC12346060; doi:10.1016/j.abd.2024.09.006)
Supplement: Supplementary file 1 [file mmc1.docx]

**ABD-D-24-00261_Supplementary Materials**

**SUPPLEMENTARY TABLES 1-7**

Adjusted linear regression model

| **Suplementary table 1 - Parameter Estimates** | | | | | | |
| --- | --- | --- | --- | --- | --- | --- |
| Dependent Variable: Pruritus intensity | | | | | | |
| Parameter | B | Std. Error | t | P value | 95% Confidence Interval | |
|  |  |  |  |  | Lower Bound | Upper Bound |
| [Fitzpatrickskintype=white] | -1.248 | .981 | -1.272 | .208 | -3.206 | .709 |
| [Fitzpatrickskintype=Asian] | -2.643 | 1.235 | -2.141 | .036 | -5.106 | -.180 |
| [Fitzpatrickskintype=pardo] | -1.366 | 1.049 | -1.302 | .197 | -3.460 | .727 |
| [Fitzpatrickskintype=black] | 0^a^ | . | . | . | . | . |
| Age | -.022 | .019 | -1.163 | .249 | -.060 | .016 |
| [Gender=F] | .414 | .475 | .871 | .387 | -.534 | 1.361 |
| [Gender=M] | 0^a^ | . | . | . | . | . |
| [face=y] | -.139 | .538 | -.259 | .796 | -1.212 | .933 |
| [face=n] | 0^a^ | . | . | . | . | . |
| [scalp=y] | .294 | .579 | .508 | .613 | -.861 | 1.449 |
| [scalp=n] | 0^a^ | . | . | . | . | . |
| [neck=y] | .599 | .550 | 1.088 | .280 | -.499 | 1.697 |
| [neck=n] | 0^a^ | . | . | . | . | . |
| [shoulder=y] | .751 | .614 | 1.223 | .226 | -.474 | 1.976 |
| [shoulder=n] | 0^a^ | . | . | . | . | . |
| [armpit=y] | -.033 | .573 | -.057 | .955 | -1.175 | 1.110 |
| [armpit=n] | 0^a^ | . | . | . | . | . |
| [chest=y] | .236 | .565 | .419 | .677 | -.891 | 1.363 |
| [chest=n] | 0^a^ | . | . | . | . | . |
| [abdomen=y] | .247 | .595 | .414 | .680 | -.941 | 1.434 |
| [abdomen=n] | 0^a^ | . | . | . | . | . |
| [buttocks=y] | .491 | .544 | .903 | .370 | -.594 | 1.576 |
| [buttocks=n] | 0^a^ | . | . | . | . | . |
| [groin=y] | -.093 | .633 | -.146 | .884 | -1.355 | 1.170 |
| [groin=n] | 0^a^ | . | . | . | . | . |
| [hand=y] | .504 | .575 | .876 | .384 | -.644 | 1.652 |
| [hand=n] | 0^a^ | . | . | . | . | . |
| [forearm=y] | 1.289 | .655 | 1.966 | .053 | -.019 | 2.596 |
| [forearm=n] | 0^a^ | . | . | . | . | . |
| [arm=y] | -1.046 | .737 | -1.419 | .160 | -2.516 | .424 |
| [arm=n] | 0^a^ | . | . | . | . | . |
| [thigh=y] | .011 | .613 | .018 | .986 | -1.211 | 1.233 |
| [thigh=n] | 0^a^ | . | . | . | . | . |
| [shin=y] | -.904 | .614 | -1.472 | .146 | -2.129 | .321 |
| [shin=n] | 0^a^ | . | . | . | . | . |
| [foot=y] | .399 | .564 | .707 | .482 | -.727 | 1.525 |
| [foot=n] | 0^a^ | . | . | . | . | . |
| [wholebody=y] | .625 | .531 | 1.177 | .243 | -.435 | 1.684 |
| [wholebody=n] | 0^a^ | . | . | . | . | . |
| a. This parameter is set to zero because it is redundant. | | | | | | |

Adjusted linear regression model

| **Suplementary table 2 - Parameter Estimates** | | | | | | |
| --- | --- | --- | --- | --- | --- | --- |
| Dependent Variable: total itchyQoL scores | | | | | | |
| Parameter | B | Std. Error | t | P value | 95% Confidence Interval | |
|  |  |  |  |  | Lower Bound | Upper Bound |
| [Fitzpatrickskintype=white] | 4.352 | 6.614 | .658 | .513 | -8.843 | 17.546 |
| [Fitzpatrickskintype=Asian] | -3.703 | 8.322 | -.445 | .658 | -20.305 | 12.900 |
| [Fitzpatrickskintype=pardo] | -.040 | 7.074 | -.006 | .995 | -14.153 | 14.072 |
| [Fitzpatrickskintype=black] | 0^a^ | . | . | . | . | . |
| Age | -.139 | .129 | -1.079 | .284 | -.395 | .118 |
| [Gender=y] | 8.572 | 3.202 | 2.677 | .009 | 2.185 | 14.959 |
| [Gender=n] | 0^a^ | . | . | . | . | . |
| [face=y] | 6.445 | 3.623 | 1.779 | .080 | -.783 | 13.674 |
| [face=n] | 0^a^ | . | . | . | . | . |
| [scalp=y] | -.196 | 3.901 | -.050 | .960 | -7.979 | 7.587 |
| [scalp=n] | 0^a^ | . | . | . | . | . |
| [neck=y] | 9.479 | 3.710 | 2.555 | .013 | 2.078 | 16.879 |
| [neck=n] | 0^a^ | . | . | . | . | . |
| [shoulder=y] | -2.976 | 4.140 | -.719 | .475 | -11.235 | 5.283 |
| [shoulder=n] | 0^a^ | . | . | . | . | . |
| [armpit=y] | 3.422 | 3.861 | .886 | .378 | -4.279 | 11.124 |
| [armpit=n] | 0^a^ | . | . | . | . | . |
| [chest=y] | 3.542 | 3.808 | .930 | .355 | -4.054 | 11.139 |
| [chest=n] | 0^a^ | . | . | . | . | . |
| [abdomen=y] | -.569 | 4.012 | -.142 | .888 | -8.572 | 7.435 |
| [abdomen=n] | 0^a^ | . | . | . | . | . |
| [buttocks=y] | 7.278 | 3.666 | 1.985 | .051 | -.035 | 14.591 |
| [buttocks=n] | 0^a^ | . | . | . | . | . |
| [groin=y] | -4.335 | 4.265 | -1.016 | .313 | -12.844 | 4.174 |
| [groin=n] | 0^a^ | . | . | . | . | . |
| [hand=y] | -1.579 | 3.877 | -.407 | .685 | -9.314 | 6.156 |
| [hand=n] | 0^a^ | . | . | . | . | . |
| [forearm=y] | 4.618 | 4.418 | 1.045 | .300 | -4.196 | 13.433 |
| [forearm=n] | 0^a^ | . | . | . | . | . |
| [arm=y] | -.256 | 4.967 | -.052 | .959 | -10.164 | 9.652 |
| [arm=n] | 0^a^ | . | . | . | . | . |
| [thigh=y] | -6.955 | 4.130 | -1.684 | .097 | -15.193 | 1.284 |
| [thigh=n] | 0^a^ | . | . | . | . | . |
| [shin=y] | -3.936 | 4.140 | -.951 | .345 | -12.196 | 4.323 |
| [shin=n] | 0^a^ | . | . | . | . | . |
| [foot=y] | 10.003 | 3.804 | 2.629 | .011 | 2.414 | 17.593 |
| [foot=n] | 0^a^ | . | . | . | . | . |
| [wholebody=y] | 8.694 | 3.580 | 2.429 | .018 | 1.552 | 15.836 |
| [wholebody=n] | 0^a^ | . | . | . | . | . |
| a. This parameter is set to zero because it is redundant. |  |  |  |  |  |  |
|  | | | | | | |

| **Suplementary table 3 - Parameter Estimates** | | | | | | |
| --- | --- | --- | --- | --- | --- | --- |
| Dependent Variable: EASI Overall Score | | | | | | |
| Parameter | B | Std. Error | t | P value | 95% Confidence Interval | |
|  |  |  |  |  | Lower Bound | Upper Bound |
| [Fitzpatrickskintype=white] | -8.197 | 6.832 | -1.200 | .234 | -21.781 | 5.388 |
| [Fitzpatrickskintype=Asian] | -5.967 | 8.205 | -.727 | .469 | -22.281 | 10.347 |
| [Fitzpatrickskintype=pardo] | -7.428 | 7.161 | -1.037 | .303 | -21.666 | 6.809 |
| [Fitzpatrickskintype=black] | 0^a^ | . | . | . | . | . |
| Age | -.174 | .129 | -1.341 | .183 | -.431 | .084 |
| [Gender=y] | -2.858 | 3.216 | -.889 | .377 | -9.252 | 3.536 |
| [Gender=n] | 0^a^ | . | . | . | . | . |
| a. This parameter is set to zero because it is redundant. |  |  |  |  |  |  |
|  | | | | | | |

| **Suplementary table 4 - Parameter Estimates** | | | | | | |
| --- | --- | --- | --- | --- | --- | --- |
| Dependent Variable: vIGA | | | | | | |
| Parameter | B | Std. Error | t | P value | 95% Confidence Interval | |
|  |  |  |  |  | Lower Bound | Upper Bound |
| [Fitzpatrickskintype=white] | -.629 | .415 | -1.516 | .133 | -1.454 | .196 |
| [Fitzpatrickskintype=Asian] | -.520 | .498 | -1.043 | .300 | -1.511 | .471 |
| [Fitzpatrickskintype=pardo] | -.375 | .435 | -.863 | .391 | -1.240 | .490 |
| [Fitzpatrickskintype=black] | 0^a^ | . | . | . | . | . |
| Age | -.011 | .008 | -1.455 | .149 | -.027 | .004 |
| [Gender=y] | -.091 | .195 | -.467 | .642 | -.480 | .297 |
| [Gender=n] | 0^a^ | . | . | . | . | . |
| a. This parameter is set to zero because it is redundant. |  |  |  |  |  |  |
|  | | | | | | |

| **Suplementary table 5 - Parameter Estimates** | | | | | | |
| --- | --- | --- | --- | --- | --- | --- |
| Dependent Variable: POEM Overall Score | | | | | | |
| Parameter | B | Std. Error | t | P value | 95% Confidence Interval | |
|  |  |  |  |  | Lower Bound | Upper Bound |
| [Fitzpatrickskintype=white] | -3.751 | 3.222 | -1.164 | .248 | -10.157 | 2.656 |
| [Fitzpatrickskintype=Asian] | -4.915 | 3.869 | -1.270 | .208 | -12.608 | 2.779 |
| [Fitzpatrickskintype=pardo] | -5.394 | 3.377 | -1.597 | .114 | -12.108 | 1.320 |
| [Fitzpatrickskintype=black] | 0^a^ | . | . | . | . | . |
| Age | -.027 | .061 | -.437 | .663 | -.148 | .095 |
| [Gender=y] | -.569 | 1.517 | -.375 | .708 | -3.584 | 2.446 |
| [Gender=n] | 0^a^ | . | . | . | . | . |
| a. This parameter is set to zero because it is redundant. |  |  |  |  |  |  |
|  | | | | | | |

| **Suplementary table 6 - Correlations** | | | | | |
| --- | --- | --- | --- | --- | --- |
|  | | Pruritus intensity | EASI Overall Score | POEM Overall Score | vIGA |
| Pruritus intensity | Pearson Correlation | 1 | .434^**^ | .610^**^ | .406^**^ |
|  | Sig. (2-tailed) |  | <.001 | <.001 | <.001 |
|  | N | 91 | 91 | 91 | 91 |
| **. Correlation is significant at the 0.01 level (2-tailed). | | | | | |

| **Suplementary table 7 - Correlations** | | | | | |
| --- | --- | --- | --- | --- | --- |
|  | | total itchyQoL scores | EASI Overall Score | POEM Overall Score | vIGA |
| total itchyQoL scores | Pearson Correlation | 1 | .425^**^ | .631^**^ | .436^**^ |
|  | Sig. (2-tailed) |  | <.001 | <.001 | <.001 |
|  | N | 91 | 91 | 91 | 91 |
| **. Correlation is significant at the 0.01 level (2-tailed). | | | | | |
